# Development of Electrospun Quaternized Poly(vinyl alcohol)/Poly(acrylamide-co-diallyldimethylammonium chloride) Anion Exchange Membranes for Alkaline Fuel Cells

**DOI:** 10.3390/nano15120907

**Published:** 2025-06-11

**Authors:** Asep Muhamad Samsudin, Viktor Hacker

**Affiliations:** 1 Department of Chemical Engineering, Universitas Diponegoro, Semarang 50275, Indonesia; asep.samsudin@live.undip.ac.id; 2 Membrane Research Center, Universitas Diponegoro, Semarang 50275, Indonesia; 3 Institute of Chemical Engineering and Environmental Technology, Graz University of Technology, 8010 Graz, Austria

**Keywords:** PVA, PAADDA, AEMs, fuel cells, electrospinning

## Abstract

Anion exchange membrane fuel cells (AEMFCs) have garnered significant attention for their potential to advance fuel cell technology. In this study, we developed and characterized anion exchange membranes (AEMs) composed of quaternized poly(vinyl alcohol) (QPVA) electrospun nanofiber mats with poly(acrylamide-co-diallyldimethylammonium chloride) (PAADDA) as a matrix filler for interfiber voids. The objective was to investigate the effect of varying PAADDA concentrations as a matrix filler for interfiber voids on the structural, mechanical, and electrochemical properties of QPVA-based electrospun AEMs. Membranes with various concentrations of PAADDA were fabricated and extensively characterized using FTIR, SEM, tensile strength, water uptake, swelling degree, ion exchange capacity (IEC), and hydroxide ion conductivity (σ). FTIR confirmed the successful incorporation of PAADDA into the membrane structure, while SEM images showed that PAADDA effectively filled the voids between the QPVA fibers, resulting in denser membranes. The results indicated that the eQPAD5.0 membrane, with the highest PAADDA content, exhibited the best overall performance. The incorporation of PAADDA into QPVA-based electrospun AEMs significantly enhanced their mechanical strength, achieving a tensile strength of 23.9 MPa, an IEC of 1.25 mmol g^−1^, and hydroxide conductivity of 19.49 mS cm^−1^ at 30 °C and 29.29 mS cm^−1^ at 80 °C, making them promising candidates for fuel cell applications.

## 1. Introduction

The decreasing availability of fossil energy and its significant adverse environmental impacts have prompted the urgent development of renewable energy sources. Fossil fuels, while still a dominant energy source, contribute heavily to greenhouse gas emissions, driving climate change and environmental degradation [1]. Various renewable energy technologies, such as solar, wind, and hydroelectric power, have been developed as cleaner alternatives. However, these technologies face challenges such as intermittent energy production, high initial costs, and infrastructure limitations, which can impede their widespread adoption [2].

Among the emerging renewable energy technologies, fuel cells are considered an eco-friendly alternative to conventional fossil fuels, offering high energy conversion efficiency and producing minimal emissions. Anion exchange membrane fuel cells (AEMFCs) are gaining significant attention as a promising alternative to conventional proton exchange membrane fuel cells (PEMFCs) due to their potential for cost reduction and environmental benefits. AEMFCs offer several advantages, including the use of non–platinum group metal (non-PGM) catalysts, which are more affordable and sustainable compared to the platinum-based catalysts required in PEMFCs [3,4]. This is facilitated by the alkaline environment in AEMFCs, which allows for the use of less expensive transition metals like nickel for the hydrogen oxidation reaction, thus reducing the overall cost of the fuel cell system [4]. Moreover, AEMFCs exhibit reduced fuel crossover and corrosion issues due to the counter-directional movement of fuel and hydroxide ions, which enhances the durability and efficiency of the system [3].

Anion exchange membranes (AEMs) are critical components in fuel cells, specifically designed to facilitate the conduction of hydroxide ions while simultaneously inhibiting electron transfer and serving as a barrier to fuel (e.g., hydrogen, ethanol, and methanol) and oxidant (i.e., oxygen). The development of anion exchange membranes (AEMs) for fuel cells faces significant challenges, particularly in achieving high ionic conductivity, chemical stability, and mechanical durability [5]. AEMs must efficiently transport hydroxide ions while withstanding the alkaline environment of the fuel cell, which often leads to chemical degradation of membrane materials. AEMs are susceptible to nucleophilic attack by hydroxide ions, particularly at elevated temperatures, compromising their structural integrity and performance [6]. The synthesis routes for membranes are frequently intricate, often involve the use of toxic chemicals, and require costly equipment. Additionally, water management within the membrane while maintaining structural integrity under operating conditions is a critical hurdle. These challenges are compounded by the need to develop cost-effective materials that can be produced at scale for commercial applications [7,8].

A wide range of polymers have been developed for AEMs, including aliphatic-based AEMs (e.g., poly(vinyl alcohol) [9] and poly(ethylene) [10]), aryl-ether-free AEMs (e.g., polybenzimidazole [11,12]), and poly(aryl ether)-based AEMs (e.g., poly (ether sulfone) [12,13] and poly(2,6-dimethyl-1,4-phenylene oxide) [14,15]). Additionally, anion exchange membranes (AEMs) have been developed with various newer polymer types, exhibiting diverse architectures and properties. Ryoo et al. developed poly(aryl piperidinium)-based AEMs using spirobifluorene as a branching agent, which improved phase separation between hydrophobic and hydrophilic domains, thereby enhancing both ion conductivity (~190 mS cm^−1^) and mechanical stability [16]. Zheng et al. prepared cross-linked/aggregated anion exchange membranes (AEMs) by incorporating hydrophilic Jeffamine cross-linkers and hydrophobic side chains into quaternized poly(vinyl benzyl chloride), resulting in high elongation at break, improved tensile strength, enhanced ionic conductivity (73.8–110.4 mS cm^−1^), and exceptional chemical stability due to micro-phase separation and alkali-resistant properties [17]. Wang et al. prepared a series of SEBS-C6-PIP-yPTP AEMs by combining the rigidity of p-triphenyl, the toughness of SEBS, and the stability of PIP cations, resulting in good mechanical and chemical stability [18]. Gokulapriyan et al. (2024) reported an ether-free poly(N-aryl piperidinium) (PNAP)–based AEM, where the optimized PNAP-2 membrane achieved a high hydroxide conductivity of 183 mS cm^−1^ at 90 °C due to the rigid aromatic backbone and optimized cation density [19]. Similarly, Arunkumar et al. (2024) developed a cross-linked poly(ether imide)–poly(triphenyl benzene piperidinium) (PEI–PTP) membrane, with the PEI–PTP-60 AEM attaining a hydroxide conductivity of 82.16 mS cm^−1^, demonstrating an effective combination of ionic conductivity and mechanical robustness [20]. These advancements highlight the versatility and potential of various polymer types in AEM development.

Poly(vinyl alcohol) (PVA) is increasingly recognized for its potential in anion exchange membranes (AEMs) for fuel cells, owing to its hydrophilic nature, which enhances water management and ionic conductivity within the membrane. The polymer’s chemical structure allows for modifications, such as introducing quaternary ammonium groups, which are crucial for anion exchange capabilities [21]. PVA membranes exhibit favorable properties, including good film-forming ability, flexibility, and mechanical strength, which are essential for the durability of fuel cells. Additionally, PVA is cost-effective and environmentally friendly, making it suitable for large-scale applications [22]. However, challenges remain, particularly regarding thermal and chemical stability under alkaline conditions, which can compromise long-term performance [21].

Various techniques and methods have been introduced for the preparation of functionalized polymer membranes. Solution casting is a widely used method for preparing anion exchange membranes (AEMs) in fuel cell applications due to its simplicity, ease of implementation, and ability to produce uniform membranes with controlled thickness [5]. This technique involves dissolving the polymer in a suitable solvent, spreading the solution onto a flat surface, and allowing the solvent to evaporate, leaving behind a solid membrane. Apart from solution casting, electrospinning is another method that is starting to attract attention. This technique employs a high-voltage source to induce an electric field between the spinneret and the collector. A Taylor cone emerges at the spinneret’s edge at a particular electric field intensity. After intensity overcomes polymer drop surface tension, an electrified solution jet is released from the Taylor cone. The solution jet evaporates and solidifies in the collector, forming nanofibers [23]. Electrospinning is particularly advantageous because it enables the formation of interlinked nanofibrous structures, significantly enhancing OH^−^ transfer within the membrane. This improved ion transport is crucial for applications like fuel cells, where efficient ion conductivity directly impacts performance. Additionally, electrospinning allows for the uniaxial alignment of polymer chains within the nanofibers, a feature that can substantially increase the mechanical strength of the membrane [24,25].

Although electrospinning offers many advantages, the fabrication of anion exchange membranes using this method is still relatively limited. Kırlıoglu et al. (2024) developed anion exchange membranes (AEMs) from PVDF-g-VBC using a radiation-induced grafting method combined with dual-fiber electrospinning [23]. These electrospun mats were then compressed into dense films through hot pressing. The resulting AEMs demonstrated an OH^−^ conductivity of 4.67 mS cm^−1^ at 25 °C, an ion exchange capacity of 1.35 mmol g^−1^, and a tensile strength of 24.4 MPa [23]. Samsudin et al. (2022) developed electrospun nanofiber mats utilizing quaternary ammonium poly(vinyl alcohol) (QPVA) and filled the voids with a mixture of QPVA and poly(diallyldimethylammonium chloride) (PDDA) [22]. The eQP-PDD0.5 membrane exhibited the highest hydroxide conductivity of 43.67 mS cm^−1^ at 80 °C, with a tensile strength of 24.95 MPa [22]. Duan et al. (2020) compared the performance of electrospun and cast quaternized poly(2,6-dimethyl-1,4-phenylene oxide) (QPPONF)/poly(vinyl alcohol) anion exchange membrane. The conductivity of electrospun QPPONF/PVA membranes significantly exceeded cast membranes at equivalent IEC values [26].

In this work, electrospun anion exchange membranes (AEMs) based on quaternary ammonium poly(vinyl alcohol) (QPVA) and poly(acrylamide-co-diallyldimethylammonium chloride) (PAADDA) were prepared and characterized for fuel cell applications. In this process, QPVA was used to form the electrospun fibers, while PAADDA served as the matrix material that filled the voids between the fibers. This combination effectively converted the electrospun mats into dense, cohesive membranes, enhancing their structural integrity and functional properties. PAADDA is favored for its hydrophilicity, eco-friendliness, and cost-effectiveness. A key feature of PAADDA is its quaternary ammonium functional groups. These groups effectively provide OH^−^ ions, enhancing ion conduction in anion exchange membranes (AEMs) [27]. The effect of the concentration of PAADDA as a void-filling matrix on the performance of electrospun QPVA/PAADDA anion exchange membranes was investigated.

## 2. Materials and Methods

### 2.1. Materials

**Figure 1:**
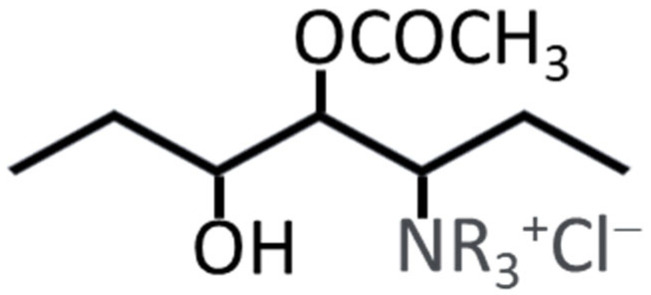
Chemical structure of Gohsenx^TM^ K-434.

Quaternary ammonium poly(vinyl alcohol) (Gohsenx^TM^ K-434, 85.5–88.0% hydrolyzed, 18–22 kDa) was sourced from Mitsubishi Chemical Corporation (Tokyo, Japan). Poly(acrylamide-co-diallyl dimethylammonium chloride) (10 wt.% in H_2_O, viscosity 9000–25,000 cP) and glutaraldehyde (25 wt.% in H_2_O) were obtained from Sigma-Aldrich (Darmstadt, Germany). Potassium hydroxide (AR), hydrochloric acid (AR), and acetone (AR) were purchased from Merck (Darmstadt, Germany). Ultrapure water (18 MΩ·cm) was produced using the Barnstead E-PURE 4-Module system. Figure 1 depicts the chemical structure of Gohsenx ^TM^ K-434.

### 2.2. Preparation of Electrospun AEMs

QPVA fiber mats were fabricated using an electrospinning technique using TL-Pro Electrospinning Equipment (Tongli Tech, Shenzhen, China). To prepare the electrospinning solution, QPVA GOHSENX™ K-434 was dissolved in ultrapure water (UPW) under continuous stirring at 80–90 °C until a 12 wt.% QPVA solution was achieved. This solution was then loaded into a 10 mL plastic syringe fitted with a spinneret needle. A high voltage of 20 kV was applied between the spinneret and a drum collector. The collector was covered with an aluminum foil substrate. The spinneret was positioned 10 cm away from the collector. The electrospinning process was carried out overnight at a flow rate of 0.5 mL/hour at room temperature with a relative humidity of 50–60%. Post-electrospinning, the QPVA fiber mats were subjected to heat treatment at 130 °C for 1 h, followed by immersion in a cross-linking solution containing 2.5 wt.% glutaraldehyde (GA) and a small amount of HCl in acetone, facilitating both physical and chemical cross-linking within the QPVA polymer chains.

**Figure 2:**
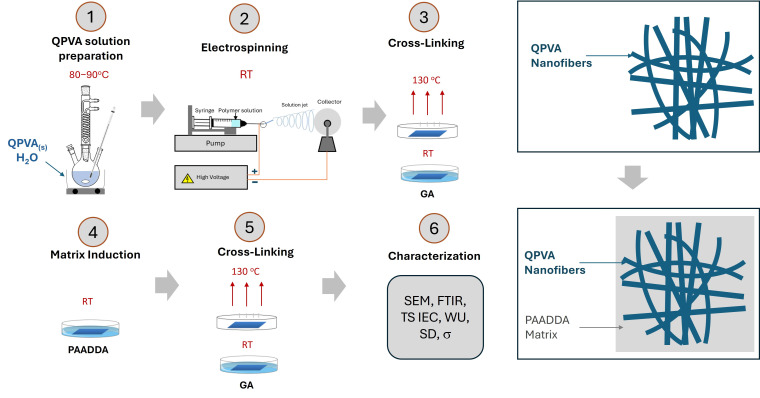
Procedure for AEM preparation.

To effectively prevent fuel crossover, it is essential to achieve a dense membrane structure by filling the voids between fibers. For this purpose, the QPVA fiber mats were immersed in PAADDA solutions of various concentrations under ambient conditions. This process led to the formation of dense anion exchange membranes. An additional cross-linking step was carried out on these dense membranes to enhance the structural integrity further and improve cross-linking between QPVA chains. The membrane samples were then designated according to Table 1. The detailed procedure for preparing these anion exchange membranes is illustrated in Figure 2.

### 2.3. Physical and Structural Characterization

The electrospun QPVA/PAADDA membranes were characterized using a variety of analytical techniques to assess their structural, thermal, and mechanical properties. Fourier Transform Infrared Spectroscopy (FTIR) was conducted using an IR-Bruker Alpha spectrometer (Bruker Optics, Ettlingen, Germany) with a resolution of 4 cm^−1^, covering a spectral range of 500 to 4000 cm^−1^, to identify functional groups and verify the presence of cross-linking within the membrane. Surface morphology and fiber structure were examined through scanning electron microscopy (SEM) using a Zeiss Supra 55VP (Carl Zeiss, Oberkochen, Germany) operated at 15 kV. Thermal stability was assessed using thermogravimetric analysis (TGA) on a STA 449 C Netsch instrument (Netsch, Selb, Germany), where samples were heated from 25 to 600 °C at a rate of 10 °C/min under a nitrogen flow of 40 mL/min. Mechanical properties, specifically tensile strength, were measured using a ZwickRoell Z010 (ZwickRoell, Ulm‑Einsingen, Germany) universal testing machine at a strain rate of 5 mm min^−1^ at room temperature, providing insights into the membrane’s durability under stress.

### 2.4. Swelling Properties Characterization

The swelling properties of the eQPAD AEMs were evaluated by measuring both water uptake and swelling degree. For water uptake, the membranes were first weighed at room temperature to obtain the initial dry weight (W_dry_). The membranes were then immersed in deionized water at room temperature for 24 h to achieve complete hydration. After removing excess surface water by gently blotting with filter paper, the membranes were immediately weighed to obtain the wet weight (W_wet_). Water uptake was calculated using Equation (1):

(1) $$Water\;Uptake(\%)=\frac{W_{wet}-W_{dry}}{W_{dry}}\times 100.$$

The swelling degree was determined by measuring the volumetric changes of the membranes before and after hydration. The initial volume (V_dry_) of the membranes was measured before immersion, and the swollen volume (V_wet_) was measured after the membranes were fully hydrated. The swelling degree was calculated using Equation (2):

(2) $$Swelling\;Degree(\%)=\frac{V_{wet}-V_{dry}}{V_{dry}}\times 100.$$

### 2.5. Ion Exchange Capacity Characterization

The ion exchange capacity (IEC) of the eQPAD AEMs was determined using a back titration method. Initially, the membranes were alkalized by soaking them in a 1.0 M KOH solution for 24 h at room temperature to convert them into hydroxide form. After thorough rinsing with deionized water to remove any unreacted KOH, the membranes were immersed in a 0.1 M HCl solution for 24 h, allowing hydroxide ions to be exchanged with chloride ions. The excess HCl that was not neutralized by the membrane was then titrated using a 0.1 M NaOH solution. This titration was performed with a Titroline^®^ 7800 automatic titrator from SI Analytics (Mainz, Germany), ensuring precise measurement of the NaOH volume required to reach the titration endpoint. The IEC was calculated using Equation (3):

(3) $$IEC(mmol\;{mg}^{-1})=\frac{(V_{a}-V_{b}){\cdot C}_{NaOH}}{W_{dry}},$$

where V_a_ and V_b_ represent the volumes of NaOH solution consumed during titration in the absence and presence of the membrane, respectively. C_NaOH_ denotes the molar concentration of the NaOH standard solution, and W_dry_ is the mass of the membrane in a dry state.

### 2.6. Hydroxide Conductivity Characterization

The hydroxide conductivity of the eQPAD AEMs was measured using electrochemical impedance spectroscopy (EIS). The membranes were first immersed in a 1.0 M KOH solution for 24 h to ensure complete conversion to the hydroxide form. Afterwards, the membranes were thoroughly rinsed with ultra-pure water (UPW) to eliminate any residual KOH. The prepared membranes were then positioned in a Bekktech BT110 LLC four-electrode conductivity clamp (Scribner Associates, Southern Pines, NC, USA), which was submerged in UPW during the measurement to maintain consistent hydration. The impedance of the membranes was measured using a Gamry Reference 600 potentiostat (Gamry Instruments, Warminster, PA, USA), operating over a frequency range of 0.1 Hz to 10 kHz with an AC amplitude of 50 mV. The membrane resistance (R_m_) was identified from the Nyquist plot by noting the intercept with the real axis (Z_real_). The hydroxide ion conductivity (*σ*) was calculated using the following equation:

(4) $$IEC(mmol\;{mg}^{-1})=\frac{(V_{a}-V_{b}){\cdot C}_{NaOH}}{W_{dry}}$$

where *d* denotes the inter-electrode distance, *T* signifies the membrane’s wet-state thickness, and *W* represents the membrane’s width.

## 3. Results

### 3.1. Chemical Structure

The FTIR spectra provide valuable insights into the chemical structure and functional group composition of the eQPAD membranes, which incorporate quaternary ammonium PVA (QPVA) fibers within a matrix of poly(acrylamide-co-diallyldimethylammonium chloride) (PAADDA). The observed spectra for the different samples, namely eQPAD_0_, eQPAD_1.0_, eQPAD_2.5_, and eQPAD_5.0_, highlight several key features across the wavenumber range of 4000 to 500 cm^−1^. First, the broad absorption bands around 3335 cm^−1^ and 3185 cm^−1^ indicate the presence of O–H stretching vibrations from hydroxyl groups in QPVA and N–H stretching vibrations from amine groups in PAADDA [28]. The increase in intensity of these bands with higher PAADDA content suggests enhanced hydrogen bonding or a greater presence of these functional groups as the PAADDA concentration increases. Additionally, the peak at 2938 cm^−1^, corresponding to C–H stretching vibrations from the aliphatic segments in both QPVA and PAADDA, shows consistent intensity across the samples, with slight variations reflecting changes in polymer content.

**Figure 3:**
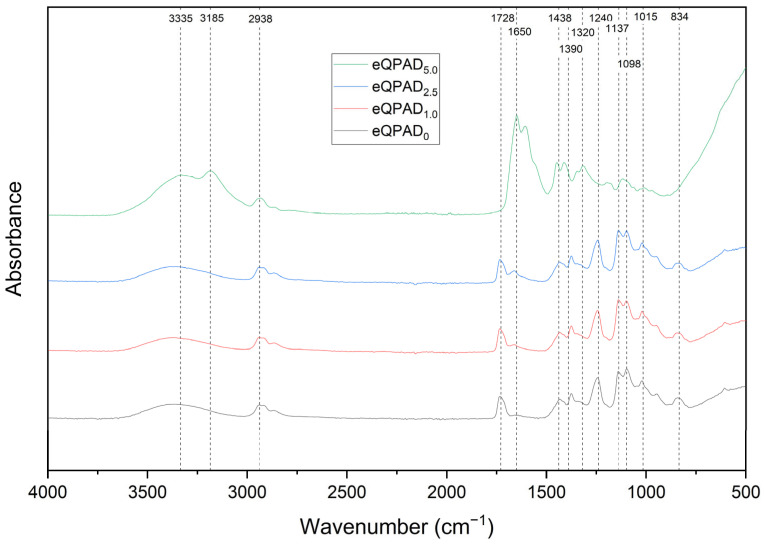
FTIR spectra of eQPAD membranes with varying PAADDA content.

The amide I and amide II bands, observed between 1728 cm^−1^ and 1650 cm^−1^, are characteristic of C=O stretching and N–H bending vibrations from the amide groups in PAADDA [28]. The variation in the intensity of these peaks confirms the presence and integration of amide functionalities within the membrane structure. Furthermore, the peaks around 1438 cm^−1^, 1390 cm^−1^, and 1320 cm^−1^ are attributed to C–N stretching and N–H bending vibrations from the quaternary ammonium groups in both QPVA and PAADDA, which become more pronounced with higher PAADDA content, reflecting the successful incorporation of these functional groups. The peaks between 1240 cm^−1^ and 1015 cm^−1^ correspond to C–O and C–O–C stretching vibrations, which are indicative of the ether linkages in QPVA. These peaks, reflecting the presence of QPVA fibers, suggest structural modifications within the membrane as PAADDA content increases. The peak at 1098 cm^−1^ corresponds to the stretching vibration of C–O bonds and –COCH_3_ groups (acetyl groups) in QPVA. The peak at 834 cm^−1^ is associated with the bending vibration of C–H bonds. As the concentration of PAADDA increases, the peaks around 3185 cm^−1^ and 1650 cm^−1^ become more pronounced, which corresponds to N–H stretching vibrations from amine groups and N–H bending vibrations from amide groups in PAADDA (See Figure 3).

### 3.2. Morphology

**Figure 4:**
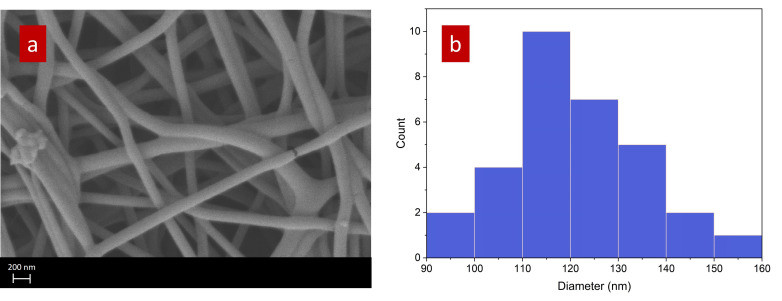
(**a**) SEM image of electrospun QPVA mats. (**b**) Diameter distribution of nanofibers.

The morphology of the electrospun QPVA nanofibers was investigated using scanning electron microscopy (SEM), as depicted in Figure 4a. The SEM image reveals a network of nanofibers with smooth surfaces and uniform morphology. The fibers are randomly oriented, creating a porous structure typical of electrospun materials. This morphology is particularly advantageous for applications in fuel cells, as the porous network can facilitate efficient gas diffusion and provide a high surface area for ion exchange processes.

Figure 4b shows the diameter distribution of the QPVA nanofibers, providing a quantitative measure of the fiber sizes observed in the SEM image. The histogram indicates that most nanofibers have diameters ranging from 100 nm to 130 nm, with a peak around 110 nm. This relatively narrow size distribution suggests that the electrospinning process was well-optimized, producing fibers with consistent diameters. Uniform fiber size is crucial in fuel cell applications, as it contributes to the consistent performance of the membrane, including its mechanical strength, ion conductivity, and overall efficiency in electrochemical reactions.

**Figure 5:**
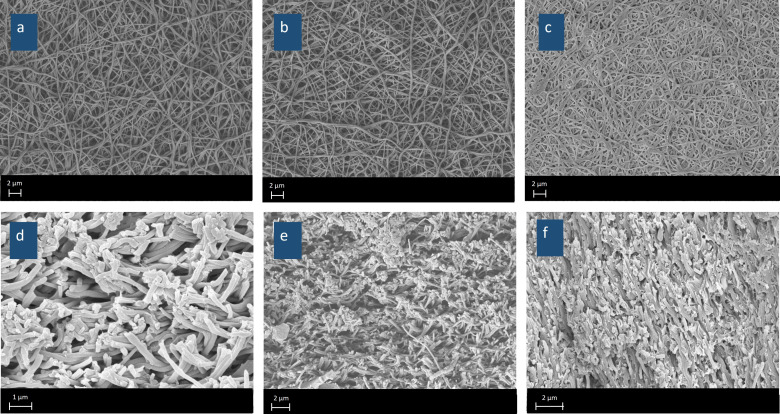
SEM images showing the surface morphology of eQPAD_1.0_ (**a**), eQPAD_2.5_ (**b**), and eQPAD_5.0_ (**c**), and the cross-sectional structure of eQPAD_1.0_ (**d**), eQPAD_2.5_ (**e**), and eQPAD_5.0_ (**f**).

Figure 5 presents SEM images illustrating the surface morphology (Figure 5a–c) and cross-sectional structure (Figure 5d–f) of eQPAD membranes with varying concentrations of PAADDA. These images provide insight into how the addition of PAADDA influences the overall density and structural characteristics of the membranes. As the concentration of PAADDA increases, the membranes become progressively denser. This densification occurs because PAADDA acts as a matrix that fills the voids between the fibers in the electrospun QPVA mat. In the surface images, eQPAD_1.0_ (Figure 5a) displays a more open fibrous structure with visible gaps between the fibers. As the PAADDA concentration increases in eQPAD_2.5_ (Figure 5b) and eQPAD_5.0_ (Figure 5c), these gaps are increasingly filled, resulting in a more compact and solidified network of fibers. The cross-sectional images further emphasize this trend. In eQPAD_1.0_ (Figure 5d), the structure is relatively open, with distinct spaces between the fiber layers. However, as more PAADDA is incorporated, as seen in eQPAD_2.5_ (Figure 5e) and especially in eQPAD_5.0_ (Figure 5f), the structure becomes denser, with fewer voids and a more uniform, tightly packed arrangement. This increased density due to the filling action of PAADDA is advantageous for reducing fuel crossover and enhancing the mechanical stability of the membrane, both of which are critical for fuel cell applications.

### 3.3. Mechanical Properties

**Figure 6:**
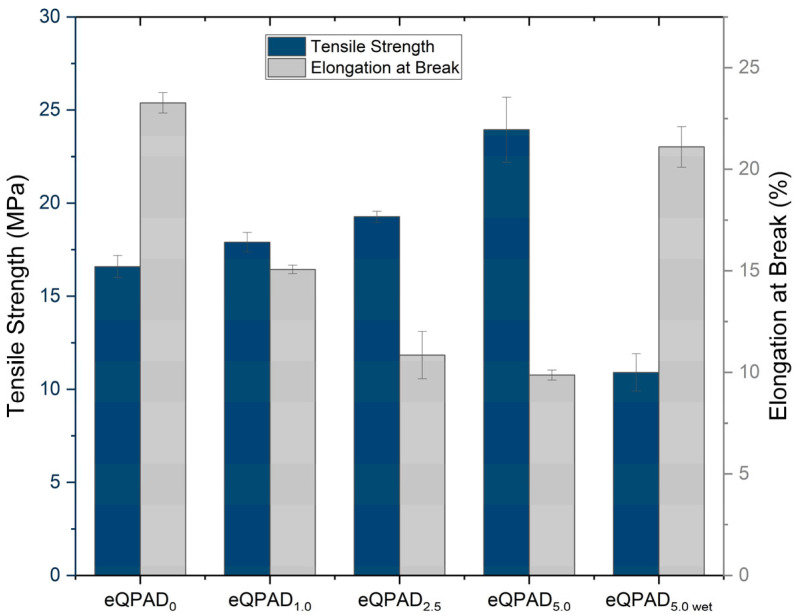
Mechanical properties of eQPAD AEMs.

The mechanical properties of the eQPAD membranes, shown in Figure 6, were evaluated in terms of tensile strength and elongation at break. These properties are crucial for assessing the structural integrity and flexibility of the membranes, especially in fuel cell applications where mechanical durability is essential. The membrane is required to possess adequate mechanical integrity to withstand the pressures encountered during both fuel cell assembly and operation.

The tensile strength of the eQPAD membranes varies with the composition. eQPAD_0_, which does not contain PAADDA, exhibits a tensile strength of 16.6 MPa. As PAADDA is introduced into the membrane (eQPAD_1.0_), the tensile strength increases slightly to 17.9 MPa, suggesting that the initial addition of PAADDA enhances the mechanical reinforcement of the membrane. This trend continues with eQPAD_2.5_, which shows a tensile strength of 19.3 MPa, and eQPAD_5.0_, which reaches 23.9 MPa. The superior tensile strength of the eQPAD_5.0_ membrane can be attributed to several factors: (i) the increased PAADDA content effectively fills the interfiber voids, leading to a denser and more compact structure that minimizes mechanical weak points [22]; (ii) the PAADDA matrix contributes additional physical cross-linking and polymer entanglement, enhancing load transfer between QPVA nanofibers [29]; and (iii) improved interfacial adhesion between fibers and matrix promotes cohesive stress distribution under tension [23]. These synergistic effects result in a mechanically robust membrane, suitable for fuel cell environments that demand structural durability and dimensional stability. Notably, the dry-state tensile strength of eQPAD5.0 (23.9 MPa) is comparable to that of commercial AEMs such as Fumatech FAA-3-50, which has a reported tensile strength of 25–40 MPa at room temperature and 50% relative humidity [30]. However, the tensile strength of eQPAD_5.0_ in its wet state drops to 10.9 MPa, reflecting a significant reduction in mechanical strength when the membrane is hydrated, which could impact its performance in a fuel cell environment.

Elongation at break, a measure of the membrane’s flexibility, decreases with increasing PAADDA content. The eQPAD_0_ AEM shows the highest elongation at break at 23.3%, indicating significant flexibility. As PAADDA is added, the elongation at break decreases to 15.1% for eQPAD_1.0_, 10.9% for eQPAD_2.5_, and 9.9% for eQPAD_5.0_. This trend suggests that while PAADDA increases tensile strength, it reduces the membrane’s flexibility, likely due to increased density and rigidity as PAADDA fills the voids between the fibers. Interestingly, when eQPAD_5.0_ is tested in its wet state, the elongation at break increases to 21.1%, indicating that hydration partially restores the membrane’s flexibility, which is advantageous in the dynamic environment of a fuel cell. The incorporation of water in AEMs induces a plasticizing effect, resulting in increased pliability and a reduction in their structural integrity [31].

### 3.4. Ion Exchange Capacity

**Figure 7:**
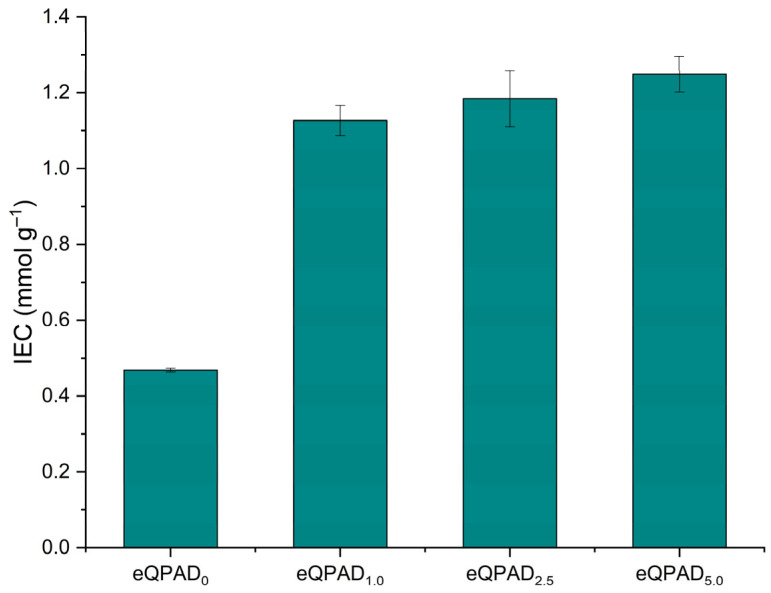
Ion exchange capacity of eQPAD AEMs.

The ion exchange capacity (IEC) is an essential characteristic of the AEMs, indicating the amount of ion-exchangeable groups within the membranes, which is vital for hydroxide conduction [32]. The ion exchange capacity (IEC) of the eQPAD anion exchange membranes (AEMs) was evaluated and is presented in Figure 7. IEC is a critical parameter that determines the membrane’s ability to conduct ions, directly influencing the performance and efficiency of fuel cells. The data illustrate a clear trend of increasing IEC values with higher concentrations of PAADDA incorporated into the membrane structure.

The baseline membrane, eQPAD_0_, exhibits an IEC of 0.47 mmol/g. This relatively low value reflects the inherent ion exchange capacity provided solely by the quaternized poly(vinyl alcohol) (QPVA) fibers without any additional modification. Upon introducing PAADDA into the membrane matrix, there is a significant enhancement in IEC. Specifically, eQPAD_1.0_ shows an IEC of 1.13 mmol/g, indicating that even a small amount of PAADDA effectively increases the number of available ion exchange sites.

As the concentration of PAADDA increases further, this trend continues, with eQPAD_2.5_ and eQPAD_5.0_ exhibiting IEC values of 1.18 mmol/g and 1.25 mmol/g, respectively. The progressive increase in IEC can be attributed to PAADDA acting as a matrix material that fills the voids between the electrospun QPVA fibers. This filling effect not only densifies the membrane structure but also introduces additional quaternary ammonium functional groups throughout the matrix. These functional groups are responsible for the ion exchange process, and their increased presence directly correlates with the enhanced IEC values observed.

The improved IEC with higher PAADDA content suggests that the membranes can facilitate more efficient ion transport, which is essential for high-performance fuel cell applications. The densification of the membrane due to PAADDA filling the inter-fiber voids also contributes to better mechanical stability and reduced fuel crossover, further enhancing the membrane’s suitability for practical use.

### 3.5. Swelling Properties

**Figure 8:**
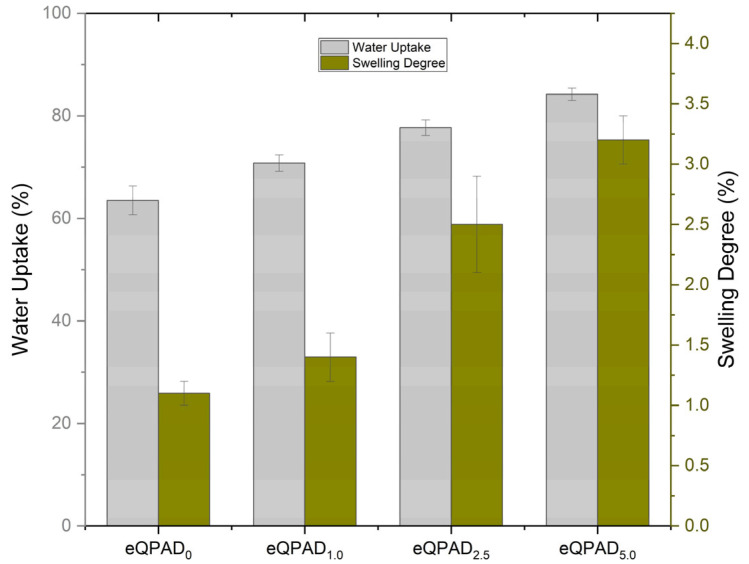
Water uptake and swelling degree of eQPAD AEMs.

Figure 8 presents the water uptake and swelling degree of eQPAD anion exchange membranes (AEMs) with varying concentrations of PAADDA. These properties are crucial for evaluating the membranes’ hydration behavior, dimensional stability, and overall performance in fuel cell applications.

The water uptake capacity of the eQPAD membranes increases progressively with the PAADDA content. The eQPAD_0_ membrane, which contains no PAADDA, exhibits a water uptake of 63.5%. This relatively high baseline value indicates that the electrospun QPVA fibers alone provide significant hydrophilicity, allowing the membrane to absorb a considerable amount of water. As PAADDA is introduced into the membrane matrix, water uptake increases further. eQPAD_1.0_ shows a water uptake of 70.8%, and this trend continues with eQPAD_2.5_ and eQPAD_5.0_, which exhibit water uptake values of 77.7% and 84.2%, respectively. The increase in water uptake with higher PAADDA content can be attributed to the hydrophilic nature of PAADDA, which enhances the membrane’s ability to absorb and retain water. This characteristic is essential for maintaining ion conductivity within the membrane, as sufficient hydration is required to facilitate ion transport.

The swelling degree, which indicates the extent to which the membrane expands upon water absorption, also increases with higher PAADDA content. The eQPAD_0_ membrane exhibits a swelling degree of 1.1%, indicating that it undergoes minimal dimensional changes upon water absorption, likely due to the relatively rigid structure provided by the QPVA fibers. As PAADDA content increases, the swelling degree rises to 1.4% for eQPAD_1.0_, 2.5% for eQPAD_2.5_, and 3.2% for eQPAD_5.0_. This trend reflects the increased flexibility and hydration capacity of the membrane as more PAADDA is incorporated. PAADDA acts as a matrix material, filling the voids between the QPVA fibers and enhancing the membrane’s ability to swell in response to water uptake. However, while higher swelling degrees can improve ion transport by increasing the free volume for ion movement, excessive swelling may compromise the mechanical stability of the membrane, potentially leading to dimensional instability under operational conditions. Noteworthy is that the maximum swelling degree observed in this study (3.2% for eQPAD_5.0_) is considerably lower than that of several commercial membranes. For instance, Tham and Kim reported a swelling ratio (SR) of FAA-3-50 ranging from 7% to 13% as the temperature increased from 30 to 80 °C, which is higher than the values specified in the manufacturer’s datasheet. In terms of water uptake, FAA-3-50 exhibited values ranging from 23 wt% to 30 wt% across the same temperature range [30]. Furthermore, Sustainion^®^ X37-50 RT, a state-of-the-art commercial AEM, demonstrates a significantly higher water uptake of over 80 wt% and an area swelling of 15.1% under hydrated conditions [30].

Compared to these commercial benchmarks, the eQPAD_5.0_ membrane developed in this study achieves a high water uptake of 84.2% while maintaining a low swelling degree of only 3.2%. This favorable combination suggests that the incorporation of PAADDA not only enhances the membrane’s hydration capacity but also effectively restrains dimensional expansion. Such a balance between water uptake and swelling behavior is critical to ensuring stable mechanical and electrochemical performance under typical fuel cell operating conditions.

### 3.6. Hydroxide Conductivity

**Figure 9:**
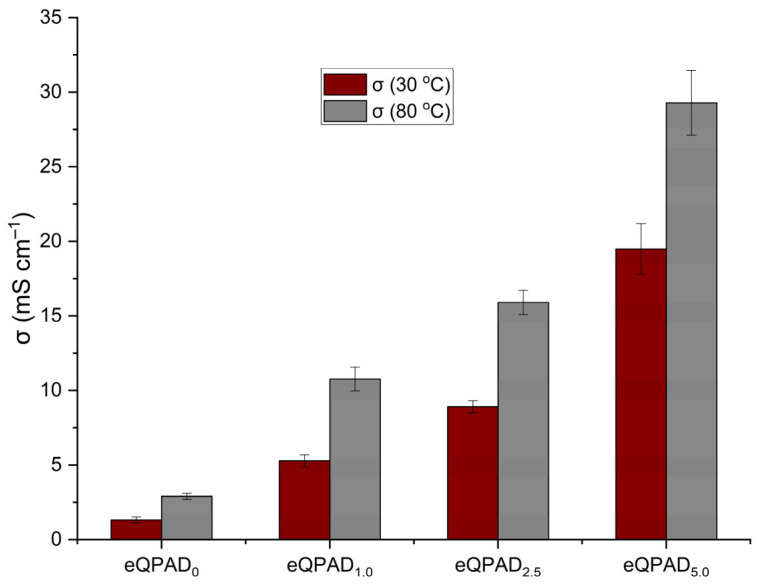
Hydroxide conductivity of eQPAD AEMs.

Figure 9 illustrates the hydroxide ion conductivity (σ) of the eQPAD anion exchange membranes (AEMs) measured at two different temperatures: 30 °C and 80 °C. The conductivity of these membranes is a critical parameter that directly impacts their performance in fuel cell applications, where efficient ion transport is essential for achieving high efficiency.

The hydroxide conductivity at 30 °C shows a clear trend of increasing values with the incorporation of PAADDA into the eQPAD membranes. eQPAD_0_, which contains no PAADDA, exhibits the lowest conductivity at 1.32 mS/cm. As the PAADDA content increases, the conductivity improves significantly. eQPAD_1.0_ reaches a conductivity of 5.29 mS/cm, while eQPAD_2.5_ achieves 8.92 mS/cm. The highest conductivity at 30 °C is observed in eQPAD_5.0_, with a value of 19.49 mS/cm. This increase in conductivity can be attributed to the enhanced ion exchange capacity (IEC) provided by the higher PAADDA content, which introduces more quaternary ammonium groups that facilitate hydroxide ion transport.

Conductivity measurements at 80 °C show a similar trend, with all samples displaying higher conductivity at this elevated temperature compared to 30 °C. eQPAD_0_ increases to 2.90 mS/cm but remains the lowest among the samples. For the PAADDA-containing membranes, conductivity rises substantially at 80 °C, reaching 10.77 mS/cm for eQPAD_1.0_, 15.91 mS/cm for eQPAD_2.5_, and an impressive 29.29 mS/cm for eQPAD_5.0_. The higher conductivity at elevated temperatures is expected, as increased temperature generally enhances ion mobility and reduces the viscosity of water within the membrane, thus facilitating faster ion transport. Moreover, an increase in temperature can facilitate the expansion of voids in AEMs, thereby enhancing ionic conductivity [33]. Compared to previous electrospun QPVA membranes that used QPVA as both nanofiber and matrix material (eQPVA_5_), which achieved only 2.26 mS/cm at 80 °C, the conductivity of the eQPAD5.0 membrane (29.29 mS/cm) represents a substantial enhancement. This improvement highlights the positive role of PAADDA in providing additional fixed ionic sites and improving membrane hydration.

The significant increase in conductivity with higher PAADDA content and elevated temperatures highlights the effectiveness of PAADDA as a matrix material that fills the voids between the electrospun QPVA fibers, thereby enhancing the overall ion transport properties of the membrane. The data indicate that eQPAD_5.0_ offers the best performance in terms of hydroxide conductivity, which is critical for fuel cell applications that require high ionic conductivity to achieve efficient operation. Table 2 summarizes the hydroxide conductivity reported for electrospun AEMs at 60–80 °C.

To further improve the conductivity of electrospun QPVA–based membranes, several strategies can be considered for future work. These include (i) increasing the degree of quaternization to enhance the ion exchange capacity [37], (ii) introducing block copolymers or inducing phase-separated domains to establish more continuous ion-conductive pathways [38], (iii) aligning nanofibers during electrospinning to promote anisotropic ion transport [39]; and (iv) incorporating inorganic ion-conductive fillers or ionic liquids to boost ionic conductivity and water retention without compromising mechanical stability [39,40].

## 4. Conclusions

This study investigated the development and characterization of anion exchange membranes (AEMs) composed of quaternized poly(vinyl alcohol) (QPVA) with varying concentrations of poly(acrylamide-co-diallyldimethylammonium chloride) (PAADDA) as a matrix material. The integration of PAADDA into the QPVA matrix was shown to significantly influence the membranes’ morphological, mechanical, and electrochemical properties, enhancing their performance for fuel cell applications. FTIR analysis confirmed the successful incorporation of PAADDA into the membrane structure, with characteristic peaks indicating the presence of quaternary ammonium groups and other functional groups associated with both QPVA and PAADDA. Morphological analysis via SEM revealed that the inclusion of PAADDA led to a denser membrane structure by effectively filling the voids within the electrospun QPVA fiber network. This densification resulted in a significant improvement in mechanical properties, with the maximum tensile strength observed in the eQPAD_5.0_ membrane reaching 23.9 MPa. However, this increase in tensile strength was accompanied by a reduction in elongation at break, from 23.3% in eQPAD_0_ to 9.9% in eQPAD^5.0^, indicating a trade-off between rigidity and flexibility that must be managed for optimal performance. Electrochemical analysis showed a clear correlation between PAADDA content and ion exchange capacity (IEC), with the maximum IEC of 1.25 mmol/g recorded for eQPAD_5.0_. This enhanced IEC contributed to the membranes’ improved hydroxide ion conductivity, particularly at elevated temperatures. The maximum conductivity observed was 19.49 mS/cm at 30 °C and 29.29 mS/cm at 80 °C for eQPAD_5.0_, indicating its potential for efficient ion transport in fuel cell applications. Water uptake and swelling degree, critical indicators of membrane hydration and dimensional stability, were also found to increase with PAADDA content. The highest water uptake reached 84.2%, and the highest swelling degree was 3.2% in the eQPAD_5.0_ membrane. While these enhancements support improved ion conductivity, the associated increase in swelling emphasizes the need for careful optimization to ensure dimensional stability under operational conditions. The eQPAD_5.0_ membrane, in particular, demonstrated the highest tensile strength, ion exchange capacity, and hydroxide ion conductivity.

Although the mechanical and hydration-related properties of the eQPAD membranes, particularly eQPAD_5.0_, are promising and comparable to commercial AEMs in terms of tensile strength, water uptake, and swelling degree, the hydroxide conductivity remains lower than that of benchmark membranes used in commercial alkaline fuel cells. Furthermore, the absence of fuel cell performance testing introduces uncertainty regarding the membranes’ practical applicability. Therefore, future research should focus on enhancing ionic conductivity through increased functional group density or phase-separated morphologies and validating the membranes’ viability through in situ fuel cell performance evaluations.

In conclusion, the incorporation of PAADDA into QPVA-based AEMs significantly enhances their mechanical and electrochemical properties, making these membranes potential candidates for fuel cell applications. While these membranes show strong potential for alkaline fuel cell applications, further optimization and performance validation are necessary to meet the stringent criteria for practical deployment.

## Figures and Tables

**Table 1:** Composition and labeling of QPVA/PAADDA AEMs.

Sample Names	Matrix Concentration (PAADDA)
eQPAD0	-
eQPAD1.0	1.0 wt.%
eQPAD2.5	2.5 wt.%
eQPAD5.0	5.0 wt.%

**Table 2:** Reported IEC and OH^−^ conductivity for PVA-based AEMs.

Materials	σ (mS·cm^−1^)	References
ASD-PAEK-X	74.00 (80 °C)	[34]
QPVA	42.00 (60 °C)	[35]
eQP-PDDA_0.5_	43.67 (80 °C)	[22]
PVA/CS	10.00 (25 °C)	[36]
PVDF-g-VBC	5.58 (25 °C)	[23]
eQPVA_5_	2.26 (25 °C)	[25]
eQPAD_0.5_	29.29 (80 °C)	This work

## Data Availability

The data supporting this article have been included as part of the manuscript.
